# Safety and usability of oncology information systems – a systematic review

**DOI:** 10.3389/fonc.2024.1231757

**Published:** 2024-11-28

**Authors:** Nikola Cihoric, Elena Riggenbach, Paul Martin Putora, Fabio Dennstädt

**Affiliations:** ^1^ Department of Radiation Oncology, Inselspital, Bern University Hospital, and the University of Bern, Bern, Switzerland; ^2^ Department of Radiation Oncology, Kantonsspital St. Gallen, St. Gallen, Switzerland

**Keywords:** electronic health record, usability, safety, oncology, oncology information systems

## Abstract

**Background:**

Digitalization has become integral to healthcare and specifically cancer treatment. Healthcare providers in oncology widely use electronic data collection and management systems in contemporary clinical practice. However, some reports point to severe problems regarding usability, which negatively impact the clinical workforce, as well as safety issues related to electronic health records, which potentially endanger patients. This systematic review aims to evaluate current scientific literature on the usability and safety aspects of electronic health records in oncology.

**Methods:**

We conducted a systematic literature review on PubMed and Scopus to identify articles on the usability and safety aspects of electronic health records and clinical information systems utilized in oncological care. Two authors independently evaluated available literature according to predefined inclusion and exclusion criteria. Finally, we quantitatively analyzed and classified the reported findings.

**Results:**

Our literature research strategy retrieved 1032 articles. We included seven articles for further processing. Three projects focused on the assessment of electronic health record functionality, three projects described the process of user-driven design, and one article described remediation procedures introduced to compensate inability of computer systems to adapt to user requirements.

**Conclusion:**

Literature on the usability and safety of electronic health records in oncology is sparse. It is essential to raise awareness and start systematic activity to promote research on the usability of electronic health records in oncology.

## Introduction

Several research groups have recently expressed severe concerns regarding the use of electronic health records. The most frequently reported issues were lack of interoperability (1), lack of standards, poor standards, poor implementation (2), or safety and usability issues (3–5). This directly results in quantifiable problems of data management, where the essential issues from the perspective of the clinician and researcher are duplication of clinical documentation (6), pressure to document negative findings (7), and failure to find data when and where needed, due to missing data or incorrect data entries (8). This situation severely affects healthcare workers and may result in direct patient harm (3, 9). In addition, a high-quality data foundation is a *conditio sine qua non* for the newest technological approaches, such as machine learning and artificial intelligence. Despite significant data-cleaning investments and other preparation processes, multiple promising technical solutions fell short when applied to broader patient cohorts (10).

The abovementioned investigations and published scientific articles are based primarily on Clinical Information Systems (CIS) and general electronic records and are not specific to oncology. However, oncology is recognized by domain specialists and international standardization groups as a complex clinical domain requiring a specialized information system profile (11–15). The need for such dedicated systems is rooted in the inherent complexity of the oncological clinical environment and the nature of information systems. A comprehensive definition of an information system includes a combination of software, hardware, and telecommunication networks to collect, process, store, and distribute valuable information. Furthermore an information system, per definition, includes formal and sociotechnical aspects of an organization (16). This implies a need for a complex interdisciplinary approach to design and development, including technical and non-technical elements (17–19).

Information systems are designed and developed based on functional and non-functional requirements. Non-functional requirements (NFR) do not relate to the specific functionality of a software system. Instead, NFR describe the system properties as a whole (20). There are six essential types of NFR, namely usability, safety, availability, scalability, effectiveness, and testability (20, 21). From the aspect of the clinicians and other healthcare workers, usability and safety interfere most with clinical routine (4, 5, 15, 22). Therefore, we conducted a systematic literature review to evaluate the current knowledge on the usability and safety of clinical software systems in oncology.

## Materials and methods

### Formulation of a problem and definition of methodology

To find relevant publications on the usability for clinical work and safety of Oncology Information Systems (OIS), we defined the following question based on the PICO framework (23): “Are there original research articles published in the medical literature analyzing the usability or safety of clinical information systems or electronic health records in oncology?”. To answer this question, we conducted this systematic review according to PRISMA guidelines for systematic reviews and meta-analysis (24).

### Formulation of definition and search criteria

For this work, we defined software usability as “the quality of a user’s experience when interacting with products or systems” and safety as “freedom from those conditions that can cause death, injury, occupational illness, damage to or loss of information or data”. In addition, we defined oncology as a clinical field involving specialties and subspecialties focused on the prevention, screening, diagnosis, treatment, and follow-up of patients with neoplastic diseases. Furthermore, we defined an OIS as an information system developed for collecting, processing, storing, and distributing information for oncological clinical practice. Under this definition, we included all software for the general collection and management of patient data within routine clinical practice, such as electronic health records (EHR) or CIS (25). Finally, for the sake of discussion, we will consider a syntagm OIS as a synonym for both EHR and CIS. We conducted literature research without limitation to any time frame.

### Eligibility criteria

Our eligibility criteria for the selection of relevant articles were as follows:

#### Inclusion criteria

- Original research articles evaluating or quantifying the usability of OIS for clinical work.
- Original research articles evaluating or quantifying safety issues of OIS.
- Original research articles describing the development of OIS or isolated OIS features with a focus on safety or usability.
- Original research articles about the implementation of measures intended to solve usability or safety issues of OIS.
- Only articles published in peer-reviewed journals were accepted.
- Only original research article published in English.

#### Exclusion criteria

- Another type of article than the original research article.
- Articles focusing on patient-oriented information systems (Electronic Patient-Reported Outcome Measures PROMs and similar).
- Articles focusing on documentation systems not intended for use in daily-clinical life (such as cancer registries).
- Articles not (or not primarily) addressing documentation of medical data from cancer patients.
- Reports analyzing IT solutions not strictly fulfilling the abovementioned definition of an OIS (e.g., software not intended for recording of data, but for processing/utilizing data recorded in EHRs (such as Clinical Decision Support Systems externally integrated into a pre-existing EHR)).
- Articles focusing on aspects other than clinical usability or safety of an OIS (including analyses of a specific data set regarding completeness or accuracy).
- Evaluation of a single feature of an OIS (e.g., Dose Prescription of Chemotherapy or radiation therapy).

### Literature search

We conducted a literature search on two bibliographic platforms, PubMed and Scopus. Based on predefined definition and criteria, we executed the following query to search for publications containing keywords in the title or abstract published within the last ten years:

*(“health record”[Title/Abstract] OR “oncology information system”[Title/Abstract] OR “clinical information system”[Title/Abstract]) AND (“usability”[Title/Abstract] OR “useful”[Title/Abstract] OR “safety”[Title/Abstract] OR “safe”[Title/Abstract] OR “security”[Title/Abstract] OR “quality”[Title/Abstract] OR “qualitative”[Title/Abstract]) AND (oncology[Title/Abstract] OR cancer[Title/Abstract]) AND (y_10[Filter])*.

We executed the literature search on 20^th^ of January 2023.

### Selection process

Review of eligible articles was done from 20 January 2023 to 10 March 2023 by two authors (NC and FD). Articles were first screened by assessing the title and the abstract for relevance to the topic based on the eligibility mentioned above criteria. Afterward, we analyzed the full text of the preselected articles. Finally, the two authors (NC and FD) discussed the eligibility of articles in cases of discrepancies or doubt.

## Results

**Figure 1:**
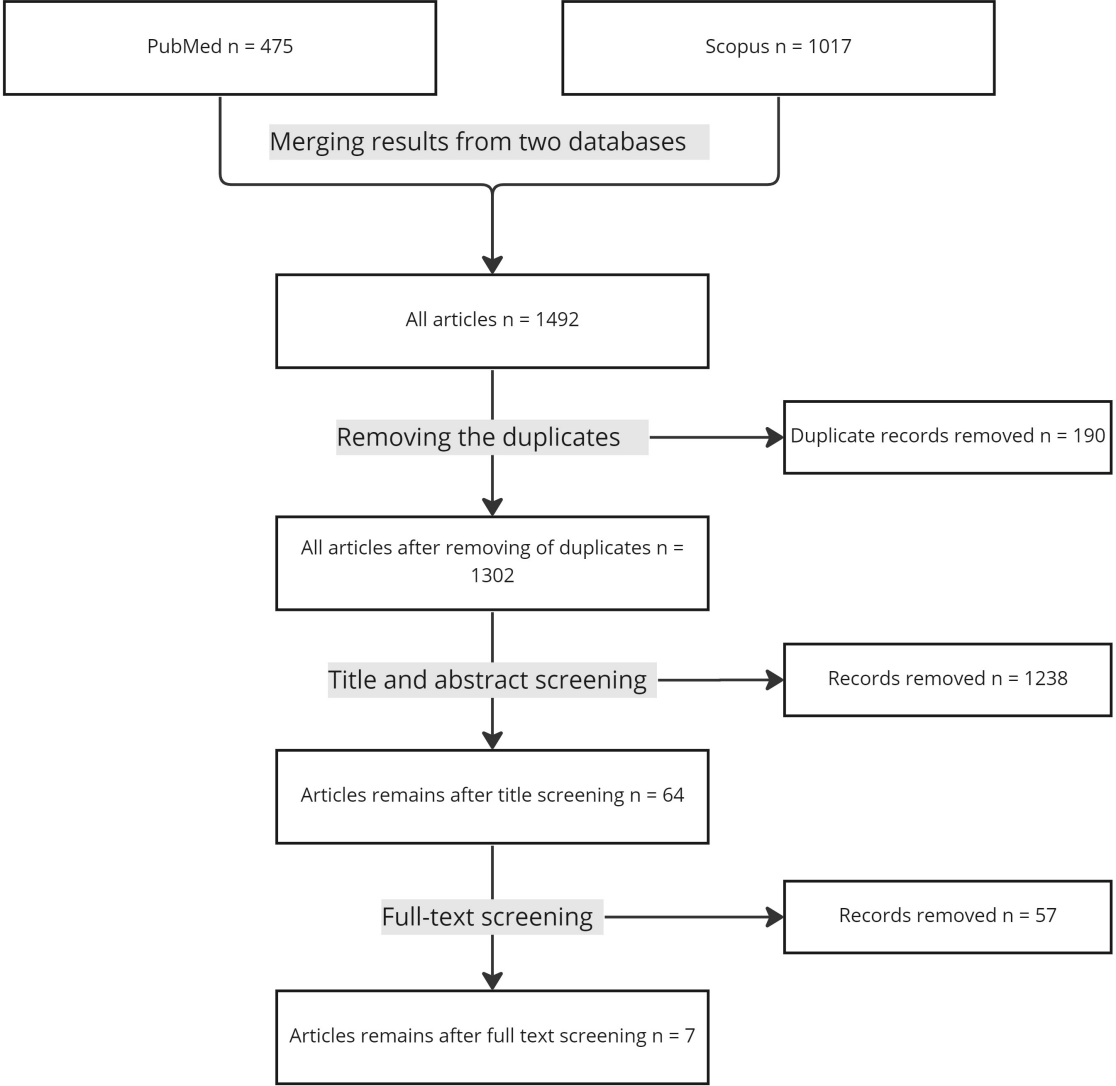
PRISMA Flowchart of the included studies.

The initial search strategy retrieved 475 articles from the PubMed database and 1017 within Scopus. After deleting duplicate records, we selected 1302 abstracts for a title and abstract screening. The title and abstract selection process revealed 64 articles eligible for full-text review. After a full-text review, the authors selected seven publications for inclusion in the study. We presented details of the selection process in **Figure 1**.

Selected articles were published in the period from 2013 to 2022. The authors of five articles focused on evaluating the usability of electronic systems. One was focused on the enhancement of safety, and one was on the enhancement of usability. All of the five publications evaluating the usability of electronic systems used a survey methodology for examining the user experience. Four of them furthermore used some sort of time measurement methodology for evaluating usefulness and practicability. One publication included the percentage of medical consultations with filled clinical notes as indicator for documentation and system usage.

Two author groups described the information systems from an ear, nose, throat (ENT) cancer center perspective, two from a general oncology perspective, one focused on breast cancer, one on pediatric oncology, and one on supportive care.

The software domain (as defined by the authors) was EHR in 4 publications. The domain of the other three publications were defined as CIS, training software and presentation dashboard.

We summarized the results of the review in **Table 1**.

**Table 1:** Summary of the systematic review.

FIRST AUTHOR	PUBLICATION YEAR	REASON FOR INCLUSION	CLINICAL DOMAIN	SOFTWARE DOMAIN AS DEFINED BY AUTHOR	KEY FINDING
**Urda D. et.al.**	2013	Evaluation of Usability	General Oncology	EHR	**Prior analysis of clinical activities and workflows, the use of adequate technology, and the availability of data analysis tools positively impact the development of OIS.**
**Jens M**	2014	Evaluation of Usability	ENT Cancer	EHR	**The central access to the information within a modern and structured user interface saves valuable time for the physician.**
**Ali B**	2018	Evaluation of Usability	Breast Cancer	CIS	**Breast-care-specific CIS positively impacts efficiency.**
**Ji EH**	2020	Evaluation of Usability	General Oncology	EHR	**Time spent on usage positively impacts data entry performance. For example, the usage of structured data entry is faster compared to free-text entry.**
**Kirk DW**	2020	Enhancement of Safety	Pediatric Hemato/Oncology	Training Software	**Interventions such as training may reduce errors connected with using computerized systems, but they cannot replace system-based solutions.**
**Chelsea LM**	2021	Enhancement of Usability	Supportive Care	Presentation Dashboard	**The design of a user-centered aggregated view of patient preferences, built on top of the existing HER solution, is feasible.**
**Tom E**	2022	Evaluation of Usability	ENT Cancer	EHR	**The introduction of computerized HER significantly prolongs clinicians’ time spent on data entry.**

### Relevant findings of selected papers

Urda D et al. described developing a web-based oncology information system designed and developed for the medical oncology department at the “Hospital Universitario Virgen de la Victoria” in Málaga, Spain, with subsequent evaluation by the users (26). The most important findings in the post-implementation review were increased data accessibility and patient progress updates. Additionally, medical practitioners perceived that OIS improved the quality of care. However, those findings contrast other objectively measured studies on general EHR, which reported adverse impacts on the patient-physician relationship to some extent. The most frequently reported problems were increased doctor computer preoccupation (27–29), affected communication flow, and challenges in verbal communication (27, 30, 31).

Meier et al. (32), from Leipzig, Germany, described developing and evaluating an EHR for data management in the ENT cancer domain called Oncoflow. A team established a set of development goals with a focus on usability. Authors compared Oncoflow on several parameters considering usability with an existing system based on the SAP(r) software. Although documentation time in Oncoflow was slightly longer, users’ evaluation reveals high satisfaction with functionalities, meeting expectations, and satisfaction with anamnesis and clinical examination modules.

Ali B at al (33). from Sydney, Australia, reported the development of a CIS specialized in breast cancer care driven by the clinical community based on agile methodology. Implementation teams set a focus of development on usability, efficiency, and adaptability. Post-implementation evaluation revealed an overall time-saving gain of 30% compared with existing enterprise EMR (Type and manufacturer not mentioned in the manuscript).

Ji EH et al. (34) from Asan Medical Center, Seoul, Republic of Korea, reported on developing structured templates for routine data collection during regular clinical care and evaluated the time and effort needed for data collection. A central finding was overall excess in the time required for documentation at the beginning, up to 70%. However, as users become more efficient after a learning period, this resulted in time gains from 1 minute and 23 seconds up to five minutes for data entry of a pathology report.

Kirk DW et al. (35), Mayo Clinic, United States, implemented a simulation-based environment with the goal of remediation of potential serious medical mistakes during a computerized order entry of chemotherapy in pediatric oncology. The study was motivated by the inability of a software vendor to adapt and change an implemented solution. Within a study, eight participants identified and mitigated an average of 5.5 out of 10 safety risks during the initial simulation and 7.4 out of 10 during a follow-up simulation.

Chelsea LM et al. (36) from Memorial Sloan Kettering Cancer Center and Weill Cornell Graduate School of Medical Science aimed to develop a usability and accessibility-focused EHR feature to enhance access to information about patient’s values and preferences. As stated by the authors, it was the first study to use a design methodology for addressing patient values for all stages of cancer care.

Tom E. et al. (37) from Radboud University Medical Center, Nijmegen, and Antoni van Leeuwenhoek Department of Head and Neck Surgery Amsterdam, Netherlands, conducted a study to quantify the time and effort spent on the EHR in ENT cancer. The authors used a cross-sectional, time-motion methodology. The results indicate significant time investment in EHR-related tasks, where 44% of initial oncological consultation and 31% of follow-up consultation duration was spent on EHR-related tasks.

## Discussion

The two most critical non-functional requirements of computer systems, usability and safety, directly impact the daily work of all stakeholders of the oncology enterprise. Despite the importance of the topic and its direct implication on patient safety, health workers’ well-being, and rising healthcare costs, the data on the usability and safety of OIS is scarce. Understanding problems encountered by the clinical workforce may help shape future-oriented information systems in oncology and other clinical disciplines.

Literature on the safety and usability of EHR is scarce. There are several possible reasons for this situation. A possible consequence of the gap in evidence is a lack of appropriate methodologies, challenges in developing relevant metrics, and prohibitive licensing rules of software providers (“gag clauses” (38).) For example, a review by Rawani et al., published in 2016, reveals a general lack of data and awareness in the clinical and scientific community (4). The cited work of Rawani et al. motivated a multi-centric study, which confirmed the hypothesis of limited usability and safety of EHRs in the ambulatory emergency medicine domain (5). A recent Swiss publication on the efficiency and safety of electronic health records, directly motivated by the work of Rawani et al., tested two EHR systems where the scenarios included internal medicine specialists engaged in clinical oncology (3). The results reveal that it is basically impossible for practicing clinicians to finish their tasks error-free. However, the major drawback of the cited papers is a one-sighted view of the clinical practitioners and their interaction with computerized systems. The authors conducted the studies from the perspective of one practician and one clinical setting. It is common sense to argue that the situation in oncology is more complex and requires specific considerations. Diagnostic requires integration of a data form multiple sources (clinical imaging, pathology, laboratory, specialists investigations). Treatment requires frequently combination of surgery, radiation therapy, and medical oncology, were significant effort must be invested in organization and management. Moreover, the most crucial decision is usually made within multidisciplinary rounds.

Consequently, physicians must coordinate numerous substeps, which follow after centralized decision-making. In addition, they must report on changes to the agreed protocol and register the overall patient state and specific outcome of clinical interventions. Moreover, this relates not only to acute or subacute care but to more than 50% of patients on longitudinal care, which frequently spans decades after the treatment (39). Undoubtfully, such an environment adds a layer of complexity to the usability and safety of computerized systems.

Based on the result of the review, we may derive several hypotheses and actionable points. First, all selected articles within the discussion pointed to the necessity of closer involvement of users in the planning, design, and implementation of computerized systems for oncology. Second, flexibility and the possibility to continuously evaluate systems for usability and safety and implement changes are necessary. Third, there is very little data on the interdisciplinary aspects of software design, development, and evaluation in oncology.

### Study limitations

We conducted a literature review on two bibliographic databases. Additional articles may be available through other sources. However, the two utilized databases cover the most relevant journals and reports with the most relevant publications. As in any review article, inclusion and exclusion criteria are a potential source of bias. To reduce this bias, we narrowly define our inclusion criteria and broadly define what should not be included. Such a strategy helps us to focus on one particular domain. Furthermore, an inherent bias is caused by the naming convention of the software utilized to manage clinical data. Focus on OIS has potentially excluded some articles assessing general EHR/CIS solutions, also covering the oncology domain to some extent. However, as stated in the introduction, we believe that the nature of oncological care requires dedicated systems, and it has specific usability and safety requirements.

## Conclusion

This review shows a lack of specific data about OIS usability and safety in oncology. The results of our systematic review did not generate sufficient evidence for best practices and guidance on future development. However, the review is valuable for raising awareness about this critical topic, as the findings of individual publications indicate considerable issues regarding usability and safety of IT systems. Further research with systematic evaluation of EHRs and CIS is needed to better understand and overcome the existing issues of impaired safety and usability.

## Data Availability

The original contributions presented in the study are included in the article/supplementary material. Further inquiries can be directed to the corresponding author.
